# The Toxicological Analysis and Toxicological Risk Assessment of Chosen Elemental Impurities (Ag, Au, Co, Cr, Cs, Li, Mo, Se, and Sr) in Green Tea (*Camellia sinensis* (L.)) Infusions

**DOI:** 10.3390/nu15061460

**Published:** 2023-03-17

**Authors:** Adrian Frydrych, Maciej Noga, Justyna Milan, Elżbieta Kondratowicz-Pietruszka, Mirosław Krośniak, Kamil Jurowski

**Affiliations:** 1 Laboratory of Innovative Toxicological Research and Analyses, Institute of Medical Studies, Medical College, Rzeszów University, Aleja Majora W. Kopisto 2a, 35-959 Rzeszow, Poland; 2 Department of Regulatory and Forensic Toxicology, Institute of Medical Expertises, Aleksandrowska 67/93, 91-205 Łódź, Poland; 3 Department of General Chemistry, Cracow University of Economics, Sienkiewicza 5, 30-033 Kraków, Poland; 4 Department of Food Chemistry and Nutrition, Medical College, Jagiellonian University, Medyczna 9, 30-688 Kraków, Poland

**Keywords:** novel impurities, elemental profile, ICP-MS, health risk assessment, green tea, tea infusions

## Abstract

Our study’s objective is to evaluate the potential health effects of elemental impurities (Ag, Au, Co, Cr, Cs, Li, Mo, Se, and Sr) found in green tea infusions (*Camellia sinensis* (L.)). The ICP-MS-based methodology was employed for elemental analysis and a detailed health risk evaluation based on weekly consumption (μg/L of infusion/week). The provisional tolerable weekly intake (PTWI), established by the Joint FAO Expert Committee on infusion/week/month based on existing literature data, was then compared to the subjects with data from the available literature. The exposure of the study items to Co ranged from 0.07904 to 0.85421 μg/day. On the contrary, the ICH (International Council for Harmonisation of Technical Requirements for Pharmaceuticals for Human Use) guidelines state that the established permitted daily exposure PDE (oral exposure) for Co is 50 μg/day. The PDE of lithium is around 560 μg/day, and according to our study, the estimated daily exposure of the evaluated products to Li fell between 0.0185 and 0.7170 μg/day. Our research also revealed modest concentrations of Cs (0.399–2.654 μg/L), Mo (0.0113–0.107 μg/L), and Sr (9.848–22.331 μg/L) in infusions. The recognized PDE for molybdenum is approximately 3400 μg/day. Only two samples contained silver, and when considering daily consumption, the predicted daily exposure to Ag is between 4.4994 and 0.03122 μg/day. The amounts of all evaluated elements in a daily dose of green tea infusions should not harm the consumer’s health. Further considerations should take aspects, such as constant change and environmental pollution, into account.

## 1. Introduction

People have been using plant extracts for health purposes for centuries, especially in the form of tea, due to their easy and accessible administration and the possibility of applying them in various situations [1]. These characteristics make tea the second most consumed beverage globally, after water [2]. Green tea was the first to be discovered. It contains large amounts of polyphenols and caffeine (2–5%, depending on the age of the leaf), as well as minerals and trace amounts of vitamins, amino acids, and carbohydrates [3]. Because green tea is not fermented, it contains much more natural substances in its fresh leaves and shows fewer vitamin losses than other types of tea [4]. Green tea has health-promoting effects due to its high content of polyphenols in fresh leaves, especially flavanols, which account for 30% of the dry weight [5]. Furthermore, green tea exceeds the catechin content of all known dietary sources, such as apples, red grapes or chocolate [6]. The catechins in tea provide beneficial effects, and (-)-epigallocatechin-3-gallate (EGCG) is the most abundant catechin [7]. According to the Food and Drug Administration (FDA), 71 mg of epigallocatechin gallate is present per 100 mL of green tea [8]. Pure epigallocatechin gallate is less stable than green tea extracts, related to their presence of antioxidant components [9]. Herbal medicines are often complex mixtures of various compounds that act synergistically with each other, allowing their beneficial health-promoting effects to be fully understood [10]. The chemical ingredients in green tea provide many health benefits for humans, such as protection against degenerative diseases due to catechins [11]. Catechins in green tea have been linked to the prevention of many types of cancer [12]. The beneficial effect of reducing the risk of many chronic diseases is attributed to the presence of powerful antioxidants (polyphenols) in green tea [13]. Green tea consumption has also been proven to lower blood sugar levels [14]. Both green tea and its extracts effectively prevent oxidative stress [15] and neurological problems [16]. In addition, green tea prevents hepatotoxicity and has antiproliferative effects on hepatoma cells [11]. Consumption of green tea may also lower blood pressure and reduce the risk of ischemic heart disease and stroke [17].

When it comes to green teas, beneficial medicinal properties are especially considered, while the problem of toxicological contamination of the soil in plant cultivation is neglected. Tea grown in polluted soil absorbs elemental ions through the roots, including heavy metals (such as Hg, Cd, and Pb), essential elements (such as Cr, Mo, and Se) and others not as well studied (such as Ag, Au, Co, Cs, Li, and Sr). These elemental impurities (EIs) are then transported to the surface and bioaccumulate in the above-ground sections of plants [18]. These impurities enter the human body after consuming such a plant or its extracts, for example, tea. Some pollutants will degrade, while the rest may accumulate in the human body. For this reason, it is worth paying attention to the significant content of these, because in excessively high concentrations, they can threaten human health [19,20]. A significant problem for the environment and substantial threat to human health is new elemental impurities (NEIs). NEIs do not result from actual environmental exposures, as in the case of conventional EIs (e.g., Co, Mo, Se, Sr, Cs, and Li). Metallic nanoparticles, such as Ag and Au, for which more information is required regarding their environmental levels and fate, are of the most important hot topics related to NEIs [21]. Excessive unintentional absorption or deliberate intake of silver compounds (usually silver dust or colloidal silver) can cause symptoms of argyria [22]. The main symptom is a change in skin colour to blue or bluish grey in places exposed to the sun’s rays, due to the deposition of silver [23]. Gold is not considered a nutrient. People are exposed to it through food chains or as a food colourant. The excess gold consumed is absorbed into the circulation and metabolised in the kidneys, liver, lymph nodes, spleen, bones, salivary glands, and bone marrow. The toxic risk associated with excess Au intake was negligible, especially compared to other metals that cause more clinically severe problems [24]. The term “EIs” (elemental impurities) in this paper describes contamination with all elements. The term “new elemental impurities” (NEIs) describes low-level elements with a harmful effect on/for the environment (e.g., Ag and Au). Traditional or conventional EIs define elements commonly found in the environment, such as the essential elements for human health and heavy metals widely studied for their high toxicity, or non-metals. Environmental EIs (Co, Cr, Cs, Li, Mo, Se, and Sr) tend to have never been evaluated because of their challenging nature and importance in the minor league. Cobalt is a vital element of vitamin B_12_; however, no specific biological functions of this element have been identified in the human body [25]. In selective hydrogenation processes, cobalt compounds are used as catalysts. Therefore, they are a possible source of this environmental element [26]. Excessive cobalt intake has been correlated with systemic toxicity, which refers to the toxic effect due to the absorption and degradation of the substance acting throughout the body and not locally, i.e., in an area distant from the point of entry. This is evidenced by a clinical syndrome with varying neurological, cardiovascular, and endocrine symptoms [27]. On the other hand, the cobalt deficit is also potentially dangerous, leading to pernicious anaemia [28]. Chromium toxicity depends on the state of oxidation. Chromium is absorbed through both the inhalation and oral pathways. Trivalent chromium is a vital mineral for human nutrition [29]. If a significant amount of chromium(III) appears in living cells, there may be a potential threat of genotoxicity. However, regular metabolism and cell function prevent it. Persistent exposure to trivalent chromium leads to weight loss, anaemia, liver failure, and renal failure [30]. On the other hand, caesium naturally occurs primarily in the form of ^133^Cs isotopes. Caesium is an exciting, but undervalued element, with 11 other major radioactive isotopes that can harm humans [31]. Caesium side symptoms include cardiac arrhythmias, hypokalaemia, fainting, convulsions, and cardiac arrest. There is no complete understanding of caesium functions [32]. Lithium is another engaging element that is used as a therapeutic agent for humans. An example is lithium salts, used to treat mania, recurrent unipolar depression, and affective problems in people with bipolar disorder [33]. An excess of lithium increases the chances of hypothyroidism and polyuria, a condition in which the body excretes excessive amounts of urine, decreased weight gain, and hyperparathyroidism [34]. In the case of toxicity associated with molybdenum consumption, there is little difference between animals treated with toxic concentrations and those that are molybdenum deficient. Exceeding the range triggers respiratory symptoms and increases neutrophils and lymphocytes [35]. Therefore, it is crucial to precisely control molybdenum content in the diet [36]. Selenium is a crucial trace element for the existence of many species and ensures the proper functioning of enzyme systems. Its most important function is to create a powerful antioxidant, an enzyme called glutathione peroxidase, which protects red blood cells and cell membranes from the damaging effects of free radicals [37]. Continuous overexposure to selenium can lead to chronic intoxication called selenosis, and is characterised primarily by epidermal and neurological effects that involve unpredictable gait and paralysis [25]. Since strontium is treated by the body similarly to calcium, it can be incorporated into the structure of the bone. Most strontium taken up is quickly excreted, and 20–30% is retained in the skeletal system [38]. Due to easy absorption and permanent incorporation into the body, radioactive isotopes from waste products of nuclear technologies are particularly dangerous, mainly ^90^Sr, which persists in a contaminated environment for a long time due to its half-life of nearly 29 years [39]. These isotopes can be inhaled with dust, although they enter the body mainly through food. It may increase the risk of bone cancer and leukaemia [40]. Our article aimed to identify and evaluate the toxicological risks to humans of novel and traditional elemental impurities (Ag, Au, Co, Cr, Cs, Li, Mo, Se, and Sr) in green tea infusions (*n* = 12) accessible in the Polish market. The first step in our studies was to determine the investigated EI and present the raw results obtained as an EI profile. The weekly consumption of green tea was then estimated (µg/L of infusion/week), and the weekly intake was assumed to be approximately 6 L of tea per week according to [41]. The next step was to estimate the weekly consumption per body weight (µg/L of infusion/week/bw). Based on the weekly intake of weekly green tea per person (~70 kg bw) compared to the provisional tolerable weekly intake (PTWI), established by the Joint FAO/WHO Expert Committee on Food Additives (JECFA). Furthermore, an individual toxicological risk assessment was performed for elements that do not include PTWI values.

Green tea is among the most consumed single-ingredient teas worldwide [25]. Despite the widely studied beneficial effects of green tea, some contaminants in tea leaves may lead to human health risks when drinking tea. In the context of exposure to green tea, this problem seems very basic, but is extremely important due to the lack of a comprehensive human toxicological risk assessment (TRA) within scientific works of NEIs and traditional EIs in green tea. Only one article on this topic in the scientific literature refers to infusions of green tea [42]. Therefore, we decided to estimate the exposure to NEIs and traditional EIs listed in the final tea infusions, and assess the safety of drinking green tea and the associated health risks. For this purpose, complete and well-designed TRA of the listed elements in green tea infusions have been developed.

## 2. Materials and Methods

### 2.1. Samples

From June 2022 to September 2022, green tea samples (*n* = 12) were purchased from stores in Rzeszów, Kraków, Toruń, Gdańsk, and Poznań in Poland. The samples analysed came in various forms, such as raw materials (in the form of leaves or needles) and tea bag containers (20–25 pieces per box; 1.4–2.0 g of raw materials). Samples were coded in a random order (GT1, GT2, etc.). Table 1 shows the characteristics of the examined green tea samples. To minimise potential impurities (elemental impurities require a specific condition in the laboratory) from other sources, all sampling procedures were performed at the analytical and clinical purity in the Bioelement Laboratory of the Collegium Medicum of the Jagiellonian University in Kraków. Furthermore, plastic equipment was applied to avoid impurities during the study. Additionally, laboratory glass equipment (volumetric flasks) was kept overnight in a 10% solution of nitric acid (HNO_3_), rinsed with distilled water, and dried in the air before analysis. Additional processing (e.g., homogenisation and digestion) was not required, because all samples were liquid samples (tea infusion). Consequently, in situ analysis was applied at the measurement stage.

### 2.2. Chemicals

In this research, nine elements (Ag, Au, Co, Cr, Cs, Li, Mo, Se, and Sr) were analysed, and two multi-element stock solutions (CHECL 01.13632.0100 and Merck 01.10580.0100) containing Ag (10.0 mg/L), Au (10.1 mg/L), Co (20.0 mg/L), Cr (20.1 mg/L), Cs (10.0 mg/L), Li (19.8 mg/L), Mo (19.9 mg/L), Se (101.0 mg/L), and Sr (9.5 mg/L), were applied as internal standards. Nitric acid (65%) was obtained from Merck (Lowe, NJ, USA).

### 2.3. Instrumentation and Determination of Elements

The determination of elements (Ag, Au, Co, Cr, Cs, Li, Mo, Se, and Sr) was performed using an ICP-MS method, that uses argon gas (plasma) to convert the sample into the ionization state for elements that are then separated, measured and investigated using a mass spectrometer. In our study, we applied an Elan DRC-e spectrometer (PerkinElmer, Waltham, MA, USA) [43]. We used simultaneous multi-element detection mode. The plasma excitation power was 1150 W; the plasma gas, carrier gas, and composition gas flow rates were 15.0 L/min, 1.1 L/min, and 1.0 L/min, respectively. All experimental conditions are summarised in Table 2. All details regarding analytical calibration and quality control are described in Supplementary Materials S1 (SM S1).

### 2.4. The Procedure of the Study

#### 2.4.1. The Green Tea Infusion Process Procedure

The green tea infusion process was carried out according to the information in Table 1 (infusion process, raw materials, and infusion time). First, an appropriate amount of green tea was poured into a beaker, and then ultrapure, demineralized, boiling water was poured over it (according to the manufacturer’s recommendations). The mixture was then covered for 3 to 8 min (according to the tea manufacturer’s recommendation), to ensure sufficient immersion [44]. After the injection, the solution was decanted and chilled to room temperature until determination.

#### 2.4.2. Toxicological Risk Assessment

For a complex toxicological risk assessment, an appropriate strategy, consisting of three crucial steps, was applied. Table 3 shows the characteristics of the applied toxicological risk assessment. This strategy was based on our previously published article on mint tea [45].

### 2.5. Statistical Analysis

Data were analysed, and graphs were generated using the OriginLab 2010 statistical software. Data processing and basic descriptive calculations, compilation, and storage of the collected data at the laboratory stage were done using Excel 2010 (Microsoft Office), licensed by Rzeszów University. The results of five independent replications are expressed as relative standard errors (RSD, %).

## 3. Results

### 3.1. The NEI and Traditional EI Profiles of All Investigated Green Tea Samples

**Figure 1:**
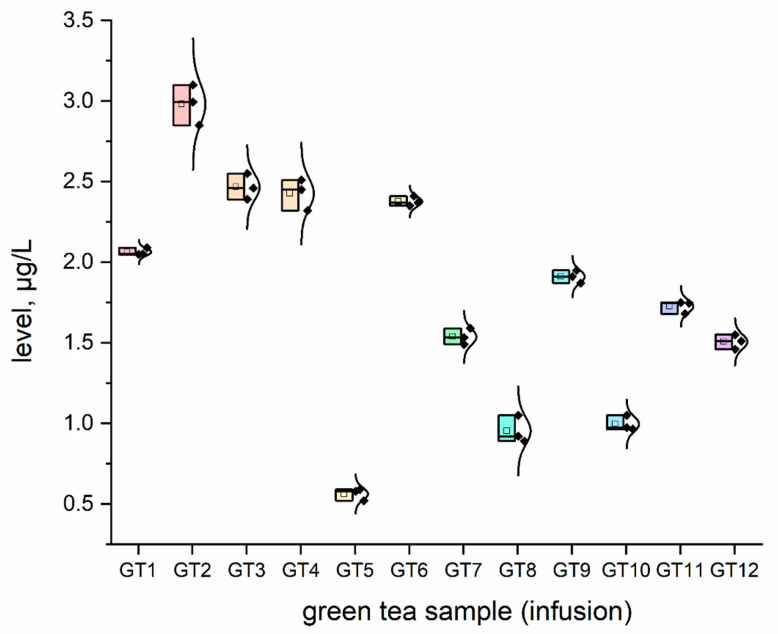
The plot as a box chart, with a normal distribution curve for Co concentration (μg/L) in analysed green tea samples (infusions; GT1–GT12). The colors indicate the results for the individual tested samples, the symbol indicates the standard deviation.

**Figure 2:**
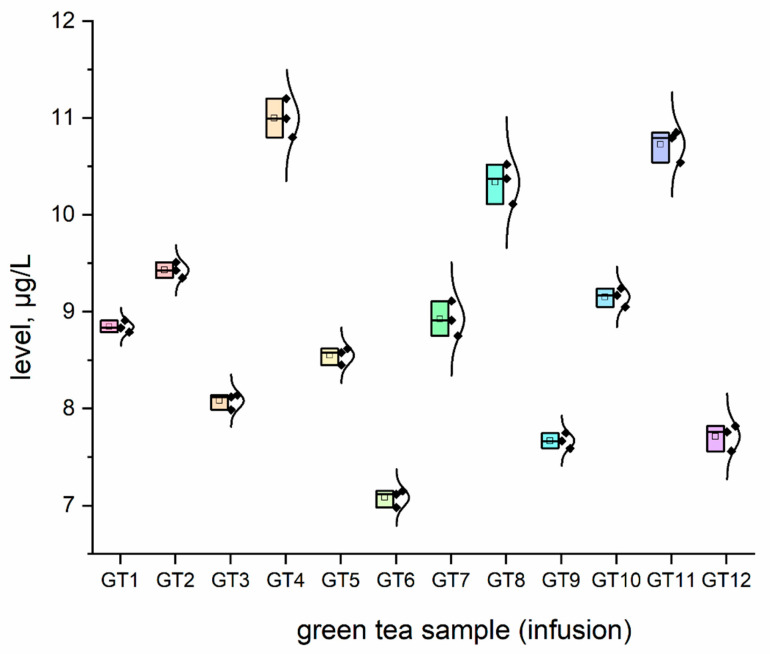
The plot as a box chart, with a normal distribution curve for Cr concentration (μg/L) in analysed green tea samples (infusions; GT1–GT12). The colors indicate the results for the individual tested samples, the symbol indicates the standard deviation.

**Figure 3:**
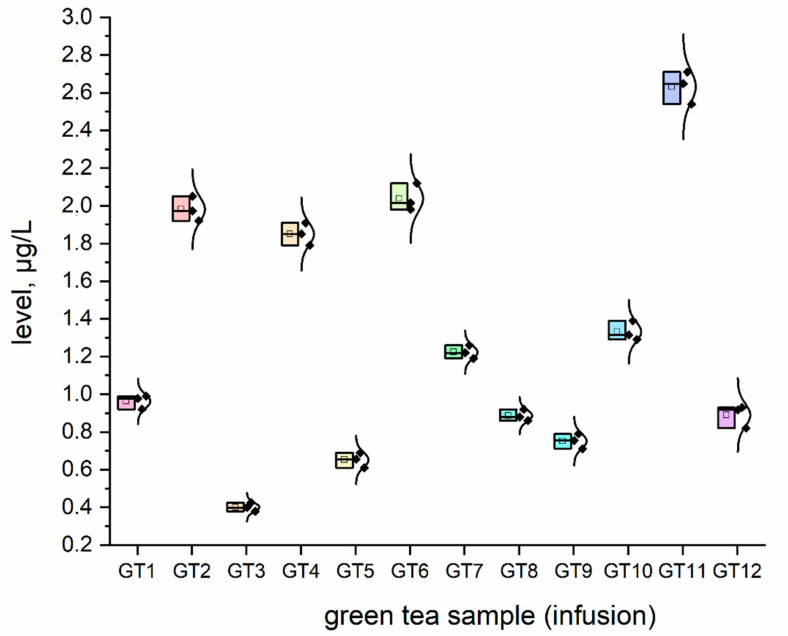
The plot as a box chart, with a normal distribution curve for Cs concentration (μg/L) in analysed green tea samples (infusions; GT1–GT12). The colors indicate the results for the individual tested samples, the symbol indicates the standard deviation.

**Figure 4:**
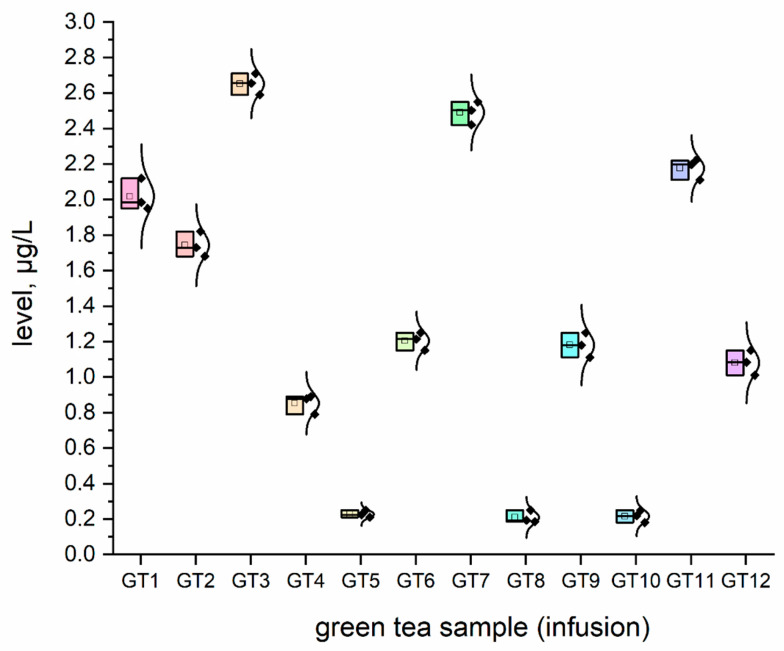
The plot as a box chart with, a normal distribution curve for Li concentration (μg/L) in analysed green tea samples (infusions; GT1–GT12). The colors indicate the results for the individual tested samples, the symbol indicates the standard deviation.

**Figure 5:**
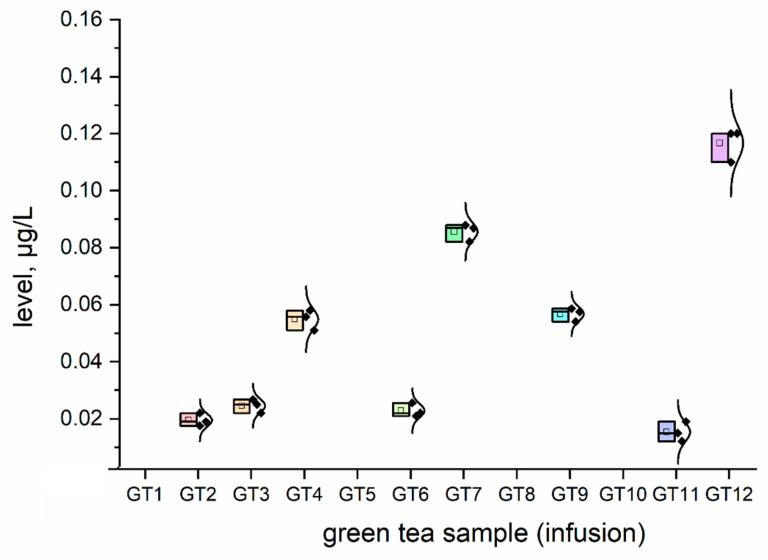
The plot as a box chart, with a normal distribution curve for Mo concentration (μg/L) in analysed green tea samples (infusions; GT1–GT12). The colors indicate the results for the individual tested samples, the symbol indicates the standard deviation.

**Figure 6:**
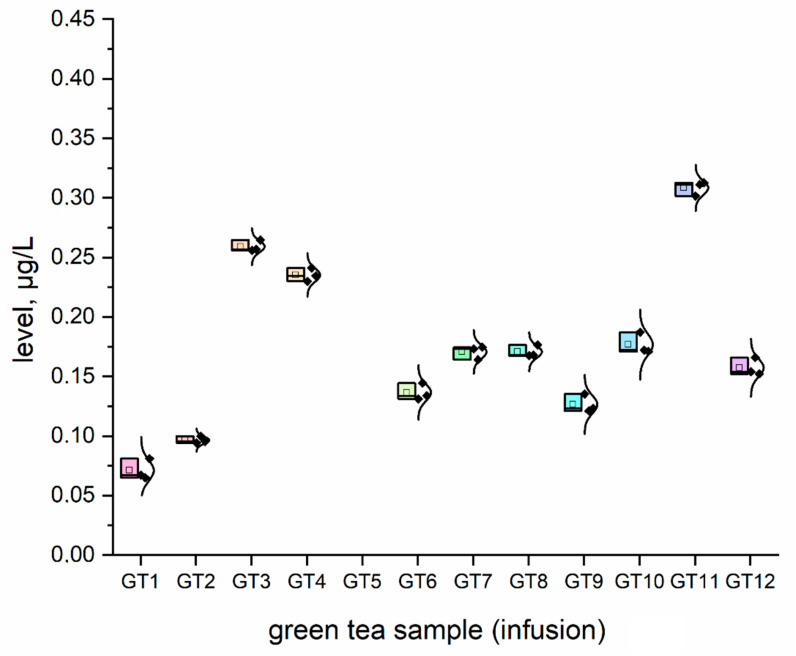
The plot as a box chart, with a normal distribution curve for Se concentration (μg/L) in analysed green tea samples (infusions; GT1–GT12). The colors indicate the results for the individual tested samples, the symbol indicates the standard deviation.

**Figure 7:**
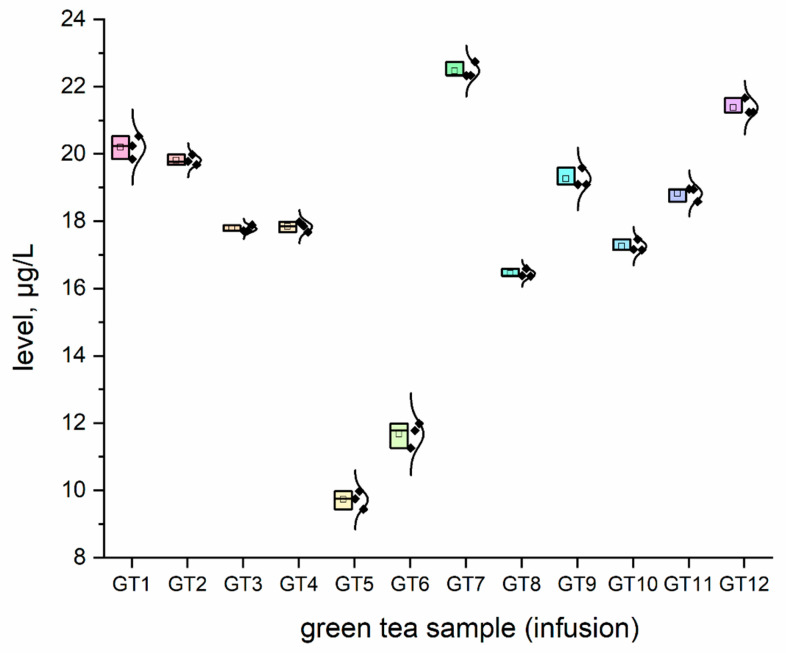
The plot as a box chart, with a normal distribution curve for Sr concentration (μg/L) in analysed green tea samples (infusions; GT1–GT12). The colors indicate the results for the individual tested samples, the symbol indicates the standard deviation.

The concentration (μg/L) of all investigated elemental impurities (Ag, Au, Co, Cr, Cs, Li, Mo, Se, and Sr) in all samples (*n* = 12; GT1–GT12) are shown in Figure 1, Figure 2, Figure 3, Figure 4, Figure 5, Figure 6 and Figure 7 as NEI and traditional EI profiles by plots, as box diagrams. All the green tea samples analysed generally contained investigated elements at different concentrations. The descriptive statistics (minimum, maximum, mean, RSD) are shown in Table 4.

All analysed green tea infusions contained all elemental impurities except silver. This element was present only in two samples; that is, GT1: 0.364 ± 0.05 μg/L and GT3: 15.748 ± 0.09 μg/L. Surprisingly, the Ag concentration in the GT3 sample was enormously high (15.748 ± 0.09 μg/L). In seven samples (GT1, GT2, GT3, GT4, GT5, GT9, and GT12), Au was present at relatively low concentrations (0.105–0.0830 μg/L). Figure 1 shows that Sr (9.848–22.331 μg/L), and Cr (7.121–10.993 μg/L) had the highest concentrations across all samples. Co (2.989 ± 0.07 μg/L), Cs (2.654 ± 0.04 μg/L), and Li (2.667 ± 0.05 μg/L) had similar maximum concentrations. The lowest concentration was observed for Mo (0.0113–0.107 μg/L). Interestingly, Ag (0.364–15.748 μg/L) and Se (0.067–0.308 μg/L) had variable values. These observations are unrelated to tea-related factors (form, raw material used, brewing time, or origin).

### 3.2. The Toxicological Risk Assessment

As described in Table 3, the second step of our toxicological risk assessment was the weekly EI assessment (based on consumption scenarios). Because there are many consumption scenarios, this was difficult. The worst scenario (WC) is usually applied to toxicological risk assessments, i.e., the highest possible frequency of weekly tea consumption. Assuming that the average consumer drinks 3–10 cups of green tea daily, the weekly intake of the elements investigated from the infusion of green tea is estimated in Table 5. The last step was estimating the weekly intake based on the weight and weekly consumption of green tea. The weekly intake of each element in the investigated samples was calculated by dividing it by 70 kg (average adult weight recommended by EFSA [50]). The results obtained are shown in Table 5.

## 4. Discussion

For complex toxicological risk assessment, we have implemented appropriate strategies composed of three key steps (Table 3). The first step was the analysis of the preliminary results of the determination of the elemental impurities (EIs) of the green tea infusion (GT1–GT12) investigated as NEI, based on the ICP-MS method, with the EI profile and descriptive statistics (minimum, maximum, average). In this phase, we demonstrated that all green tea infusions (impurities profile: Figure 1, and normal distribution curve boxes: Figure 2, Figure 3, Figure 4, Figure 5, Figure 6 and Figure 7) show a relatively low concentration of EIs in all green tea infusions. The element profiles obtained in the green tea infusions revealed the presence of Ag (0.364–15.748 μg/L), Au (0.0105–0.0830 μg/L), Co (0.580–0.989 μg/L), Cr (7.121–10.993 μg/L), Cs (0.399–2.664 μg/L), Li (0.205–2.667 μg/L), Mo (0.0113–0.107 μg/L), and Se (0.067–22.331 μg/L). It should be noted that Ag was present only in two samples (GT1: 0.364 ± 0.05 μg/L and GT3: 15.748 ± 0.09 μg/L). Surprisingly, Ag concentration in the GT3 sample was enormously high (15.748 ± 0.09 μg/L). Furthermore, seven samples (GT1, GT2, GT3, GT4, GT5, GT9, and GT12) contained Au, but were relatively low (0.105–0.0830 μg/L). This article is the first in the scientific literature to determine the selected elements of the green tea infusion (raw results). We tried to compare our results (μg/L) with data from the literature on selected elements. Only one article [51] described the determination of the same elements, but only four of the elements (Co = 1.715 μg/L, Cs = 1.515 μg/L, Mo = 0.043 μg/L, Sr = 7.25 μg/L) coincided. The results are very comparable for the first three elements, and the only significant difference is in the case of strontium (in our case, Sr = 17.763 µg/L). The difference may result from comparing the average values based on the product ranges of the variables and the products analysed (*n* = 12). There are several articles in the scientific literature in which the elements were also determined. However, compared to our article, dry matter and green tea leaves were used as samples for analysis, and the data were presented in µg/g, µg/kg and mg/kg [52,53,54,55,56,57]. In the second and third steps of toxicological risk assessment, the weekly intake (μg/week) in the 600–2000 ml range, and the weekly intake per body weight depending on green tea consumption, were examined (Table 5). Since most of the EIs in the investigation (Ag, Au, Co, Cr, Cs, Li, Mo, and Sr) did not have an established value of PTWI, individual health risk assessments were evaluated. The results indicate that daily EI concentrations should not represent a health risk to consumers after consuming green tea infusions from the products available on the Polish market. The possible assessment of health risks did not show any health hazards to consumers for weekly exposure only for Se.

### 4.1. Silver

Silver is a naturally occurring element. Silver can exist in various forms, such as soluble silver compounds, insoluble compounds, or in the form of metallic silver. Soluble silver compounds can potentially cause adverse effects on the human body, because they are more easily absorbed than metallic or insoluble silver. In the example of overexposure to silver nitrate, symptoms include diarrhoea, stomach irritation, breathing problems, or a drop in blood pressure. After prolonged inhalation or ingestion of soluble silver compounds or colloidal silver, the most familiar characteristic, irreversible discolouration of the skin (argyria) and/or eyes (argyrosis), may appear [58]. With reference to the daily consumption (about 250 ml of green tea infusion daily), the estimated daily exposure to Ag was approximately 0.03122–4.4994 μg/day. According to the ICH guideline Q3D (R1) on elemental contaminants, the established permitted daily exposure (PDE and oral exposure) for Ag is 167 μg/day [59]. This means that drinking green tea is safe. Silver was present only in two samples (GT1: 0.364 ± 0.05 μg/L and GT3: 15.748 ± 0.09, μg/L).

### 4.2. Gold

Gold was present in all samples (0.105–0.0830 μg/L), but in seven at relatively low concentrations. Toxicity studies have shown that Au tends to exhibit relatively little, if any, toxicity, since many cytotoxicity studies show that gold is non-toxic [60]. The gastrointestinal tract partially absorbs elemental gold or released ions. Organs such as the liver, heart, kidneys, and lungs receive gold. Gold is primarily expelled in the urine after ingestion. There are only a few studies on the oral toxicity of elemental gold. According to Hadrup et al., rats in their investigation were unaffected by a single dose of 2000 mg of nanoparticles/kg body weight, suggesting that elemental gold has low acute toxicity [61]. According to the ICH Q3D guideline (R1), the PDE for Au of 134 μg/day, Au impurities are not hazardous to human health and are not pollutants with a crucial concentration in the environment. The study showed that you could take 0.00049–0.02074 μg/day of Au when drinking green tea (250 mL of green tea infusion per day), which is safe for health.

### 4.3. Cobalt

Cobalt has magnetic properties and promotes oxidation and reduction reactions. Exposure to cobalt and its compounds causes adverse health effects, such as carcinogenicity in humans [62]. Cobalt impurities were observed in all samples (0.580–2.989 μg/L). There was no Co assessment in the Joint FAO/WHO Expert Committee on Food Additives (JECFA) database. The established PDE (oral exposure) for Co by the ICH Q3D guideline (R1) is 50 µg/day [59]. Compared to the findings of this investigation, the Co impurities in green tea are low. Providing the body with such an amount of cobalt, by consuming the green tea available in Poland, does not threaten human health. The estimated daily exposure of the body to this element is between 0.07904 and 0.85421 μg/day (approximately 250 mL of green tea infusion per day). Co impurities are negligible compared to the 50 g/day (oral exposure) Co established PDE by the ICH Q3D (R1) guideline [59], and do not pose a risk to people.

### 4.4. Chromium

Chromium (Cr) is a “hazy” chemical element regarding human health. Although it is a crucial micronutrient, it is also linked to several diseases and toxic effects, including carcinogenicity. We still do not fully understand how Cr and its components work in humans. The chromium content in tested teas varied between 7.121 and 10.993 μg/L. Chromium PDE is described in the ICH guideline Q3D (R1), which is 10700 μg/day [59]. In the tested green teas, the Cr concentration was within the range of 7.12–10.99 μg/L. According to our estimates, drinking green tea does not pose a risk of chromium toxicity.

### 4.5. Caesium

Caesium was present in each tested sample, in concentrations between 0.399 and 2.654 μg/L. The knowledge of the metabolism and toxicity of caesium is limited [63]. Oral consumption of caesium chloride has been widely promoted based on a hypothesis called “high-pH cancer treatment” [64]. This element is excreted by the kidneys in humans. The biokinetic model gives the following percentages: urine at 85%, faeces at 13%, and sweat at 2% [65]. Taking into account the content of caesium in green tea infusions tested in Poland, its consumption appears safe for the human body. The ICH Q3D (R1) guideline and the Joint FAO/WHO Expert Committee on Food Additives (JECFA) database lack Cs papers. As a result, it is impossible to compare the data obtained, although the low concentration of this element suggests no significant health problems for humans.

### 4.6. Lithium

A popular and successful treatment for mood disorders is lithium. Although there has been concern about its safety, there is insufficient evidence for side effects. The lithium content in the tested samples varied between 0.205 and 2.667 μg/L. Lithium can be harmful if used in excess or in situations with a risk of fluid or sodium deficit. When drinking green tea, the risk of lithium toxicity is not significant [34]. Consuming green tea infusions does not pose a risk associated with the toxic effects of lithium. The established PDE (oral exposure) for Li by the ICH Q3D guideline (R1) is based on human experience with this element, and is approximately 560 μg/day. Since the expected daily exposure to Li in the items under investigation (approximately 250 mL of green tea infusion per day) ranges from 0.0185 to 0.7170 μg/day, there is no potential issue with Li exposure after green tea consumption.

### 4.7. Molybdenum

Very little is known about the effects of Mo on human health. This trace element is essential for both animals and plants. In mammals, molybdenum is present as a component of some metalloflavoproteins. Molybdenum is present in drinking water in the range of 0.11–6.2 or 0–20 μg L^−1^ [66]. Our studies found low molybdenum concentrations in green tea infusions (0.0113–0.107 μg/L). From a regulatory point of view, this element only has a PDE (oral) value of 3400 g/day [59]. No risk is associated with green tea infusions, because the Mo concentration range in the infusions tested was between 0.002177 and 0.02488 μg/day.

### 4.8. Strontium

The estimated exposure to strontium in the green tea infusions investigated available in Poland was in the range of 9.848–22.331 μg/L. Strontium had the highest concentrations across all samples. However, our results compared to the value of PDE (oral) for this element, described in the ICH Q3D (R1) guideline, which is 120 µg/day, are relatively low; therefore, strontium concentrations do not pose a health risk, including drinking green tea infusions available in Poland.

### 4.9. Selenium

Selenium is poisonous at high concentrations, and several examples of apparent toxicity and fatalities after acute poisoning have been documented [67]. The trace element selenium (Se) is essential for synthesising amino acids, such as selenocysteine and selenomethionine. We discuss the acute hazardous effects, including exposure and concentrations in the blood and urine, linked to mortality [67]. The selenium content in the tested green tea samples was in the range of 0.067–0.308 μg/L. With the regular consumption of green tea, selenium contamination does not threaten the human body health. The PTWI for selenium is 66 μg/kg bw/week, meaning drinking 600–2000 mL of green tea covered by the study is safe for the human body [68].

## 5. Conclusions

Our developed EI research health risk assessment strategy provides pioneering data (Ag, Au, Co, Cr, Cs, Li, Mo, Se, and Sr) and can be helpful for additional research and manufacturers. Furthermore, well-designed health risk assessment methods will be valuable and essential for public health and environmental studies. As these environmental studies are rare, it would be useful to conduct a broader study considering other green tea infusions in different countries, differentiating them, for example, according to their origin, supply chain, and other aspects of food production that may restrict the toxicological safety of green teas.

## Figures and Tables

**Table 1:** List and data of teas used for the impurity profile and toxicological risk assessment.

Code of Sample	Form of Tea	The Amount of Raw Material Intended for a Single Brewing Process, g	Time of the Brewing Process (Brew Time), Minutes	Country of Origin	EAN
GT1	Tea bag	1.5	1–3	Sri Lanka	5901086000333
GT2	Tea bag	1.5	3	China	5900175401532
GT3	Leaf tea	2.0	2–3	China	5900738004101
GT4	Leaf tea	2.0	3	Sri Lanka	5901483051129
GT5	Leaf tea	4–5	3	China	5907732943986
GT6	Leaf tea	3.0	4	China	5900956700410
GT7	Tea bag	1.5	1–3	China	5906881826072
GT8	Tea bag	2	2–3	Sri Lanka	4796004230449
GT9	Tea bag	2	3–4	China	5900956006782
GT10	Tea bag	1.5	1–3	Sri Lanka	4791038950158
GT11	Leaf tea	2.0	3–5	Sri Lanka	5900396000736
GT12	Tea bag	1.75	2–3	China	20321154

**Table 2:** ICP-MS operating conditions and performance.

Parameter	Value(s)
Instrument	Elan DRC-e Perkin Elmer (US)
Calibration	External *
RF power	1150
Dwell time	250 ms
Sweeps/Readings	4
Readings/Replicates	2
Replicates	3
Spray chamber	Cyclonic spray chamber
Nebulizer	Meinhard nebulizer
Cooling gas flow rate (L/min)	17
Sampler cone	Ni
Scanning mode	Peak-hopping
Plasma gas flow rate	15 L/min
Carrier gas flow rate	1.1 L/min
Composition gas flow rate	1.0 L/min

* External calibration—Because the pure and certified standard solution used in the calibration process is external to the sample, the calibration is called external standard calibration. This type of calibration can be applied successfully by comparing analytical signals from standard solutions with samples.

**Table 3:** Description of the applied toxicological risk assessment.

Step	Description
1	Analysis of raw results from the determination of investigated elemental impurities in green tea infusions (g/L of infusion) as NEI and traditional EI profiles of investigated samples (GT1-GT12) and descriptive statistics (minimum, maximum, average);
2	Estimation of weekly intake (g/L infusion/week) based on weekly tea consumption (approximately 21–70 cups of green tea infusions per week based on the review of the literature [46,47,48,49]);
3	Depending on weekly tea consumption per person compared to PTWI, weekly intake was evaluated according to body weight (µg/L of infusion/week/bw), using the equation: EWIBW = EWI/BW, where EWI is the estimated weekly intake (µg/L of infusion/week), and BW is the average body weight (approximately 70 kg bw) (kg).

**Table 4:** The descriptive statistics of examined elements in each analysed sample (GT1–GT12).

Statistical Parameter	Elemental Impurity								
	Ag	Au	Co	Cr	Cs	Li	Mo	Se	Sr
Minimum, μg/L	0.364	0.0105	0.580	7.121	0.399	0.205	0.0113	0.067	9.848
Maximum, μg/L	15.748	0.0830	2.989	10.993	2.654	2.667	0.107	0.308	22.331
Mean, μg/L	8.0560	0.0333	1.798	8.981	1.304	1.340	0.0486	0.170	17.763
RSD, %	0.334	0.00031	0.0054	0.0199	0.0058	0.00983	0.00061	0.0021	0.0157

**Table 5:** The estimation of the weekly intake (µg/week) in the range of 600–2000 mL, and weekly intake per body weight estimation of the examined elements based on the consumption of green tea.

Sample	Estimation of Weekly Intake, µg/Week								
	Ag	Au	Co	Cr	Cs	Li	Mo	Se	Sr
GT1	0.219–0.729	0.044–0.145	1.229–4.097	5.302–17.673	0.586–1.954	1.196–3.986	N/D	0.041–0.135	12.502–41.673
GT2	N/D	0.016–0.053	1.794–5.979	5.658–18.860	1.189–3.963	1.050–3.501	0.011–0.036	0.057–0.189	11.966–39.888
GT3	9.49–31.496	0.008–0.027	1.523–5.078	4.822–16.073	0.240–0.799	1.600–5.335	0.016–0.052	0.155–0.518	10.631–35.436
GT4	N/D	0.013–0.042	1.504–5.014	6.596–21.988	1.114–3.713	0.524–1.745	0.034–0.113	0.139–0.464	10.742–35.808
GT5	N/D	0.050–0.167	0.348–1.160	5.147–17.157	0.396–1.321	0.136–0.455	N/D	N/D	5.909–19.697
GT6	N/D	N/D	1.388–4.626	4.273–14.242	1.210–4.034	0.724–2.413	0.015–0.051	0.080–0.266	7.060–23.532
GT7	N/D	N/D	0.918–3.058	5.363–17.878	0.737–2.458	1.506–5.020	0.052–0.174	0.098–0.328	13.399–44.662
GT8	N/D	N/D	0.553–1.844	6.221–20.736	0.528–1.760	0.123–0.410	N/D	0.100–0.335	9.826–32.755
GT9	N/D	0.006–0.021	1.152–3.841	4.595–15.316	0.455–1.518	0.703–2.343	0.035–0.116	0.073–0.244	11.454–38.180
GT10	N/D	N/D	0.587–1.955	5.534–18.446	0.793–2.643	0.130–0.433	N/D	0.103–0.343	10.293–34.309
GT11	N/D	N/D	0.925–3.084	6.497–21.657	1.593–5.309	1.310–4.367	0.007–0.023	0.185–0.617	11.369–37.895
GT12	N/D	0.003–0.012	N/D	4.658–15.527	0.553–1.845	0.647–2.158	0.064–0.214	0.093–0.310	12.744–42.479
**Sample**	**Estimation of Weekly Intake, µg/Week/bw**								
	**Ag**	**Au**	**Co**	**Cr**	**Cs**	**Li**	**Mo**	**Se**	**Sr**
GT1	0.00312–0.01041	0.00062–0.00207	0.01756–0.05853	0.07574–0.25247	0.00837–0.02791	0.01708–0.05694	N/D	0.00058–0.00193	0.17860–0.59533
GT2	N/D	0.00023–0.00075	0.02563–0.08542	0.08083–0.26942	0.01698–0.05661	0.01500–0.05002	0.00016–0.00052	0.00081–0.00270	0.17095–0.56982
GT3	0.13498–0.44995	0.00012–0.00038	0.02176–0.07254	0.06888–0.22962	0.00342–0.01141	0.02286–0.07621	0.00022–0.00074	0.00222–0.00740	0.15187–0.50624
GT4	N/D	N/D	0.02149–0.07162	0.09423–0.31411	0.01591–0.05304	0.00748–0.02493	0.00048–0.00161	0.00199–0.00663	0.15346–0.51154
GT5	N/D	N/D	0.00497–0.01658	0.07353–0.24510	0.00566–0.01888	0.00195–0.00650	N/D	N/D	0.08442–0.28139
GT6	N/D	N/D	0.01983–0.06609	0.06104–0.20346	0.01729–0.05763	0.01034–0.03447	0.00022–0.00073	0.00114–0.00381	0.10085–0.33617
GT7	N/D	N/D	0.01311–0.04369	0.07662–0.25540	0.01053–0.03511	0.02151–0.07171	0.00075–0.00249	0.00141–0.00468	0.19141–0.63803
GT8	N/D	N/D	0.00790–0.02635	0.08887–0.29623	0.00754–0.02515	0.00176–0.00586	N/D	0.00143–0.00478	0.14038–0.46792
GT9	N/D	0.00009–0.00030	0.01646–0.05487	0.06564–0.21880	0.00650–0.02168	0.01004–0.03347	0.00050–0.00165	0.00105–0.00349	0.16363–0.54543
GT10	N/D	N/D	0.00838–0.02794	0.07906–0.26352	0.01133–0.03775	0.00186–0.00618	N/D	0.00147–0.00490	0.14704–0.49013
GT11	N/D	N/D	0.01467–0.04890	0.09282–0.30939	0.02275–0.07584	0.01872–0.06239	0.00010–0.00033	0.00264–0.00881	0.16241–0.54136
GT12	N/D	0.0005–0.00016	0.01322–0.04405	0.09282–0.30939	0.00791–0.02635	0.00925–0.03083	0.00092–0.00306	0.00881–0.00881	0.18205–0.60685

N/D—no data.

## Data Availability

The datasets generated during and/or analysed during the current study are available from Kamil Jurowski (kjurowski@ur.edu.pl) upon reasonable request.

## Supplementary Materials

The following supporting information can be downloaded at: https://www.mdpi.com/article/10.3390/nu15061460/s1, Table S1. The summary of analytical calibration strategy and quality control results.

## Abbreviations

EAN	European article number
EGCG	(-)-epigallocatechin-3-gallate
EI	Elemental impurity
FAO	The Food and Agriculture Organization
FDA	Food and Drug Administration
ICH	The International Council for Harmonisation of Technical Requirements for Pharmaceuticals for Human Use
ICP-MS	Inductively coupled plasma mass spectrometry
JECFA	Joint FAO Expert Committee on Food Additives
NEI	New elemental impurity
PTWI	Provisional tolerable weekly intake
RSD	Relative standard deviation
TRA	Toxicological risk assessment
WHO	World Health Organization
