# Supplementary material for: The Toxicological Analysis and Toxicological Risk Assessment of Chosen Elemental Impurities (Ag, Au, Co, Cr, Cs, Li, Mo, Se, and Sr) in Green Tea (Camellia sinensis (L.)) Infusions
Source: Nutrients. 2023 Mar 17;15(6):1460. doi: 10.3390/nu15061460 (PMC10053437; doi:10.3390/nu15061460)
Supplement: Supplementary file 1 [file nutrients-15-01460-s001.zip › nutrients-2249837-Supplementary Materials.pdf]

Supplementary Materials 1 (SM1)

The Analytical Calibration Strategy and Quality Control

To ensure adequate quality standards, an appropriate calibration strategy and a quality control approach have been developed. The quantitative analysis of the elements in the infusion samples investigated is carried out using an analytical calibration method (calibration curves) obtained by diluting the stock standards of the elements studied (1:100 – 1:10000). Ag, Au, Cd, Cs, Pb, and Sr concentrations (n = 5) were 0.0, 1.0, 2.0, 5.0, 10.0 mg/L, Co, Cr, Li and Mo concentrations were 0.0, 2.0, 4.0, 10.0, 20.0 mg/L. Se standard solutions were prepared, 10.0, 20.0, 50.0, and 100.0 g/L and were applied for the analytical calibration procedure. The coefficients obtained (0.996 < R < 0.999) show that the analysis is accurate and precise. Table S1 shows a summary of the analytical calibration strategy and quality control results.

Table S1. The summary of analytical calibration strategy and quality control results.

**Analyte**	**Calibration Function**		**R**	**Recovery, %**
	**Sigma A**	**Slope**		
Ag	49.3241	3141.38	0.99986	98 ± 1.0
Au	4.45668	69.3612	0.99586	97 ± 1.3
Co	10.066	8651.94	0.99999	99 ± 0.7
Cs	46.851	14,413.5	0.99988	97 ± 1.5
Li	3.80757	1006.25	0.99998	98 ± 1.1
Mo	23.9563	3380.03	0.99992	97 ± 1.5
Se	0.79798	153.457	0.99996	97 ± 1.3
Sr	115.175	16,393.8	0.99992	96 ± 1.6
